# Blood DNA methylation and COVID-19 outcomes

**DOI:** 10.1186/s13148-021-01102-9

**Published:** 2021-05-25

**Authors:** Joseph Balnis, Andy Madrid, Kirk J. Hogan, Lisa A. Drake, Hau C. Chieng, Anupama Tiwari, Catherine E. Vincent, Amit Chopra, Peter A. Vincent, Michael D. Robek, Harold A. Singer, Reid S. Alisch, Ariel Jaitovich

**Affiliations:** 1 Division of Pulmonary and Critical Care Medicine, Albany Medical Center, Albany, NY USA; 2 Department of Molecular and Cellular Physiology, Albany Medical College, Albany, NY USA; 3 Department of Neurological Surgery, University of Wisconsin, Madison, WI USA; 4 Department of Anesthesiology, University of Wisconsin, Madison, WI USA; 5 Department of Immunology and Microbial Disease, Albany Medical College, Albany, NY USA

**Keywords:** COVID-19, DNA methylation, Mortality, Outcomes, Acute respiratory distress syndrome (ARDS), Gene expression

## Abstract

**Background:**

There are no prior reports that compare differentially methylated regions of DNA in blood samples from COVID-19 patients to samples collected before the SARS-CoV-2 pandemic using a shared epigenotyping platform. We performed a genome-wide analysis of circulating blood DNA CpG methylation using the Infinium Human MethylationEPIC BeadChip on 124 blood samples from hospitalized COVID-19-positive and COVID-19-negative patients and compared these data with previously reported data from 39 healthy individuals collected before the pandemic. Prospective outcome measures such as COVID-19-GRAM risk-score and mortality were combined with methylation data.

**Results:**

Global mean methylation levels did not differ between COVID-19 patients and healthy pre-pandemic controls. About 75% of acute illness-associated differentially methylated regions were located near gene promoter regions and were hypo-methylated in comparison with healthy pre-pandemic controls. Gene ontology analyses revealed terms associated with the immune response to viral infections and leukocyte activation; and disease ontology analyses revealed a predominance of autoimmune disorders. Among COVID-19-positive patients, worse outcomes were associated with a prevailing hyper-methylated status. Recursive feature elimination identified 77 differentially methylated positions predictive of COVID-19 severity measured by the GRAM-risk score.

**Conclusion:**

Our data contribute to the awareness that DNA methylation may influence the expression of genes that regulate COVID-19 progression and represent a targetable process in that setting.

**Supplementary Information:**

The online version contains supplementary material available at 10.1186/s13148-021-01102-9.

## Introduction

More than 3 million deaths worldwide have been attributed to COVID-19, primarily arising from acute respiratory distress syndrome (ARDS). The clinical course of SARS-CoV-2 infection is highly variable, ranging from an asymptomatic state to life-threatening infection [1–4]. Recent evidence indicates that disease severity predominantly depends on host factors [5–10], supporting the need to better resolve individual responses at the molecular level. We and others have recently described the multi-omic profile of COVID-19 patients in association with disease severity [10–12]. Analysis of mRNA sequencing from circulating leukocytes identified multiple expressed genes associated with worse outcomes [10, 11, 13].

Because almost every cell in an individual shares identical genomic sequence, distinct cellular phenotypes are established and maintained by epigenetic mechanisms [14, 15], including DNA, histone and chromatin modifications and non-coding RNA expression that affect the chromatin landscape [16]. Although DNA cytosine methylation at 5′–C–phosphate–G–3′ (CpG) sites is pervasive in the genome and is considered relatively stable [15–18], it is highly sensitive to age and environmental factors [16, 19–23]. However, CpG rich regions (CpG islands) in gene promoter regions of actively transcribed genes are typically hypomethylated and selective hypermethylation of key cytosine residues may repress gene transcription by modifying transcription factor accessibility [16]. Moreover, methylation of cytosine residues outside the context of CpG sites may also affect gene transcription [16]. Critically ill patients exhibit altered circulating blood DNA methylation profiles [24, 25]. Epigenetic marks affect gene expression profiles and increase individual vulnerability to viral infections [26]. For example, modulators of host–pathogen interactions including interferons are epigenetically regulated [27, 28], and DNA methylation has been shown to underpin antigen-presentation following MERS-CoV infection [27–29]. To date, no methylomes of samples from COVID-19 patients have been compared to pre-pandemic sample sources, or to samples from patients with non-COVID-19 respiratory illness using a shared epigenotyping platform and facility. Moreover, it is currently unknown whether patients with worse outcomes and distinct transcriptomes [11, 30, 31] may be further distinguished by patterns of differential methylation. These data carry strong potential to illuminate mechanisms underlying COVID-19-associated gene expression and outcomes [32, 33], and may facilitate the identification of sub-phenotypes likely to benefit from specific interventions [34–36]. For example, immune-modulating drugs such as corticosteroids, that are beneficial in COVID-19 patients [7, 37–39], interact with gene expression-response elements throughout the genome. Resolution of the differential methylome in COVID-19 patients offers potential insights into COVID-19 pathogenesis, susceptibility, diagnosis and prognosis.

**Fig. 1:**
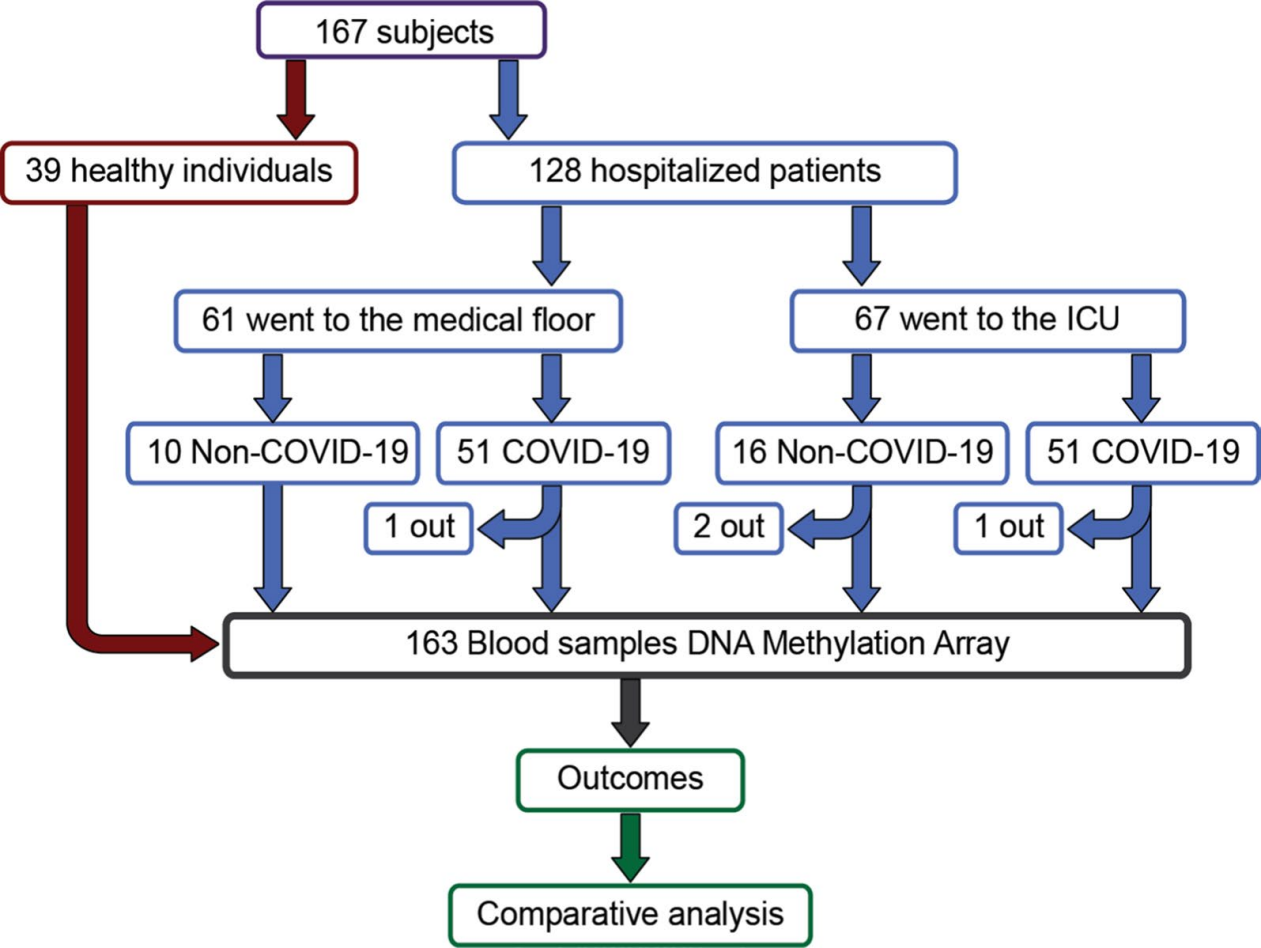
Diagram of the entire cohort involved in study: Notice that while the hospitalized patients’ cohort contributed 128 patients, *only 124* were part of the analyses due to inadequate quality of 4 samples; see diagram and details in the text

Accordingly, we conducted a prospective cohort study involving 124 consecutive patients with and without COVID-19 diagnosis who were admitted to Albany Medical Center in Albany, New York. Thirty-nine healthy patient samples collected before the COVID-19 pandemic characterized with an identical epigenotyping platform provided reference methylomes (Fig. 1). We hypothesized that: (1) DNA methylation regions would differ in patients with COVID-19 diagnosis in comparison with pre-pandemic healthy control individuals; (2) DNA methylation regions would differ in patients with COVID-19 diagnosis in comparison with patients with respiratory illness of similar magnitude not caused by COVID-19; and 3) COVID-19 severity reflected by clinically validated outcome measures [40], would be associated with distinct patterns of DNA methylation in blood.

## Results

### Sample cohort and experimental design

From 6 April 2020 through 1 May 2020, we collected blood samples from 128 adult patients admitted to the Albany Medical Center in Albany, NY for moderate to severe respiratory failure presumably related to infection with SARS-CoV-2 (Fig. 1). In addition to acquisition of various clinical data (Table 1), a 10 ml blood sample was obtained at the time of enrollment. Patients who later tested positive (*N* = 102) and negative (*N* = 26) for SARS-CoV-2 infection were assigned to the COVID-19 and non-COVID-19 groups, respectively (see “Methods” for enrollment details). Females comprised 37.3% and 50.0% of the COVID-19 and non-COVID-19 patients, respectively. The average age of patients was similar: 60.5 (50.5–74.8) and 62 (50–74) years in the COVID-19 group (females and males, respectively; *p* value = 0.28) compared the non-COVID-19 patients: 59.5 (49–75) and 68.2 (63–82) years, (females and males respectively; *p* value = 0.09). The average number of days hospitalized before study enrollment was 3 and 1 for the COVID-19 and non-COVID-19 patients, respectively (Table 1). To identify DNA CpG methylation changes associated with COVID-19, we compared DNA methylation data from COVID-19 patients (*N* = 102) to DNA methylation data from a previously published study [21] that profiled DNA methylation from whole blood of healthy participants (*N* = 39) that was collected at least 3 years before the COVID-19 outbreak. An identical epigenotyping platform and facility (Genuity Science, Inc. Boston, MA) was used to obtain the methylation data. To test whether COVID-19 severity correlates with patterns of differential DNA methylation in blood, we used the COVID-19 specific GRAM risk score [40] and patient mortality. Other clinical data included: Acute Physiologic Assessment and Chronic Health Evaluation (APACHE II) score, Sequential Organ Failure Assessment (SOFA) score [41], SAPS II score, Charlson Comorbidity Index score [42], mechanical ventilation physiological parameters, need for admission to intensive care, and C-reactive protein (CRP), D-dimer, ferritin, lactate, procalcitonin, fibrinogen, and other levels (Table 1). APACHE II, SOFA, and SAPS II severity scores assigned to patients in intensive care, exhibited similar distributions between the groups (Table 1). In keeping with previous reports, males predominated in the group requiring intensive care (66 vs. 33%) and mechanical ventilation (46.9 vs. 34.2%, Additional file 1: see clinical data Table S1).

**Table 1:** Demographics and baseline characteristics of COVID-19 and non-Covid 19 in ICU and non-ICU setting and healthy controls

Variables	COVID-19			Non-COVID-19			Healthy
	Total n = 102	non-ICU n = 51	ICU n = 51	Total n = 26	non-ICU n = 10	ICU n = 16	Total n = 39
Days admitted pre-enrollment (iqr)*	3.37 (1–5)	2.78 (1–3)	3.96 (1–6)	0.97 (1–1)	0.9 (0.8–1)	0.94 (1–1)	N/A
*Sex—n (%)*							
Male	64 (62.7%)	30 (58.8%)	34 (66.7%)	13 (50%)	4 (40%)	9 (56%)	18 (46%)
Female	38 (37.3%)	21 (41.2%)	17 (33.3%)	13 (50%)	6 (60%)	7 (44%)	21 (54%)
*Age-year*							
Mean (IQR)^+^	61.3 (50.0–74.3)	59.7 (49.0–80.0)	62.9 (55.0–73.0)	63.8 (52.3–76.8)	60.4 (47.3–74.0)	66 (55.3–80.3)	75.8 (71.9–78.8)
*Etnicity—n (%)*							
White*^+^	46 (45.1%)	28 (54.9%)	18 (35.3%)	21 (80.8%)	8 (80%)	13 (81.2%)	35 (89.7%)
Black	11 (10.8%)	5 (9.8%)	6 (11.8%)	4 (15.4%)	2 (20%)	2 (12.5%)	4 (10.3%)
Asian*^+^	2 (1.9%)	0 (0%)	2 (3.9%)	0 (0%)	0 (0%)	0 (0%)	0 (0%)
Hispanic*^+^	21 (20.6%)	7 (13.7%)	14 (27.5%)	1 (3.8%)	0 (0%)	1 (6.3%)	0 (0%)
Other*^+^	22 (21.6%)	11 (21.6%)	11 (21.6%)	0 (0%)	0 (0%)	0 (0%)	0 (0%)
*BMI, kg/m*^*2*^* mean (IQR)*	30.39 (25.30–32.24)	29.84 (26.09–32.37)	30.92 (24.50–32.05)	30.36 (26.53–33.10)	27.20 (23.68–30.38)	32.34 (26.98–37.67)	28.52 (24.15–30.40)
*Severity indexes (IQR)*							
Charlson comorbidity index*	3.3 (1–5)	3.16 (1–5)	3.49 (2–5)	4.35 (2–6)	3.3 (1–5)	5 (3–7)	N/A
APACHEII	N/A	N/A	21.6 (15–27)	N/A	N/A	20.6 (12–26)	N/A
SOFA	N/A	N/A	8.2 (6–11)	N/A	N/A	8.6 (3–11)	N/A
SAPSII	N/A	N/A	51.8 (45–62)	N/A	N/A	47.6 (33–65)	N/A
*Biomarkers (IQR)*							
Ferritin (ng/mL)*	938.9 (301.8–1203.8)	782.6 (206.0–934.5)	1076.9 (378.0–1294.0)	250.5 (80.5–382.5)	205.3 (58.0–411.0)	285.7 (92.0–438.5)	N/A
C-Reactive protein (mg/L)*	140.9 (52.0–204.3)	120.6 (44.7–155.0)	158.9 (61.7–248.3)	73.8 (20.0–110.2)	34.7 (8.9–56.8)	99.8 (37.8–175.2)	N/A
D-dimer (mg/L FEU)	11.7 (1.0–12.8)	2.3 (0.6–1.73)	18.6 (1.7–21.6)	5.3 (0.5–4.6)	5.2 (0.4–1.9)	5.5 (0.6–10.2)	N/A
Procalcitonin (ng/mL)	3.2 (0.2–1.8)	1.7 (0.2–1.0)	4.4 (0.3–2.3)	2.1 (0.2–0.7)	2.2 (0.1–3.4)	2.1 (0.3–1.21)	N/A
Lactate (mmol/ L)*	1.2 (0.9–1.5)	1.2 (0.9–1.4)	1.3 (0.9–1.5)	2.1 (0.9–2.5)	1.2 (0.8–1.5)	2.53 (0.9–3.4)	N/A
Fibrinogen (mg/dL)*	543.6 (413.0–667.0)	559.3 (420.0–703.0)	531.7 (391.5–663.0)	362.3 (257.3–550.0)	348.0 (256.75–441.5)	373 (257.3–572.0)	N/A
Albumin (mg/L)*	2.9 (2.6–3.3)	3.2 (2.9–3.5)	2.7 (2.4–2.9)	3.4 (2.9–3.8)	3.8 (3.4–4.1)	3.19 (2.6–3.8)	N/A
*Hemogram (IQR)*							
White blood cells (K/uL)	10.8 (6.1–12.5)	7.1 (4.9–8.5)	14.4 (8.4–15.4)	12.7 (7.2–17.3)	8.3 (6.7–9.7)	15.4 (8.2–20.9)	N/A
Hemoglobin (g/dL)*	11.2 (9.7–12.6)	11.6 (10.2–13.0)	10.7 (9.4–12.1)	12.4 (9.9–14.7)	12.8 (10.45–14.85)	12.3 (9.6–14.5)	N/A
Mean corpuscular volume (fL)*	87.1 (84.5–93.7)	88.0 (85.6–94.2)	86.2 (82.5–93.0)	92.3 (88.6–95.4)	91.2 (87.2–94.6)	93.0 (89.4–97.8)	N/A
Platelet (K/uL)*	266.0 (192.5–320.5)	269.2 (209.0–334)	262.8 (187.0–317.0)	203.5 (151.8–247.8)	228.1 (163.5–278.0)	188.2 (127.5–229.5)	N/A
Neutrophils (%)	76.2 (68.5–86.0)	69.7 (61.0–82.0)	82.8 (80.0–90.0)	77.7 (74.0–87.0)	73.1 (58.8–82.5)	80.5 (79.25–89.25)	N/A
Lymphocytes (%)	13.8 (5.0–18.5)	19.4 (9.0–26.0)	8.3 (4.0–11.0)	12.7 (6.0–18.0)	16.9 (7.0–26.0)	10.1 (4.3–10.8)	N/A
Monocytes (%)	7.1 (4.0–9.0)	8.8 (6.0–11.0)	5.5 (3.0–8.0)	8.0 (4.0–9.3)	7.7 (4.0–10.3)	8.2 (4.0–9.0)	N/A
Eosinophils (%)	0.8 (0.0–1.0)	1.1 (0.0–1.0)	0.5 (0.0–1.0)	1.0 (0.0–1.25)	1.8 (0.0–3.3)	0.44 (0.0–1.0)	N/A
*Respiratory parameters*							
PaO2/FiO2 Ratio	N/A	N/A	161.6 (98–211)	N/A	N/A	149.4 (73–184)	N/A
Positive-end expiratory pressure (cmH2O)*	N/A	N/A	10.8 (10–12)	N/A	N/A	6.6 (73–184)	N/A
Inspiratory Plateau (cmH2O)	N/A	N/A	22.8 (19.7–25.3)	N/A	N/A	23.9 (19.8–28.8)	N/A
*Treatment—n (%)*							
Renal Replacement Therapy	12 (11.8%)	3 (5.9%)	9 (17.6%)	3 (11.5%)	0 (0%)	3 (18.8%)	N/A
Hydroxychloroquine*	87 (85.3%)	43 (84.3%)	44 (86.3%)	0 (0%)	0 (0%)	0 (0%)	N/A
Antibiotics*	98 (96.1%)	47 (92.2%)	51 (100%)	16 (61.5%)	3 (30.0%)	13 (81.3%)	N/A
Antiviral*	1 (0.98%)	0 (0%)	1 (1.9%)	0 (0%)	0 (0%)	0 (0%)	N/A
IL6-Antagoinist*	4 (3.9%)	1 (1.9%)	2 (3.9%)	0 (0%)	0 (0%)	0 (0%)	N/A
Convalescent Plasma*	26 (25.5%)	8 (15.7%)	18 (35.3%)	0 (0%)	0 (0%)	0 (0%)	N/A
Steroid*	46 (45.1%)	12 (23.5%)	34 (66.7%)	4 (15.4%)	1 (10.0%)	3 (18.8%)	N/A
Therapeutic Anticoagulation	37 (36.3%)	2 (3.9%)	35 (68.6%)	8 (30.8%)	1 (10.0%)	7 (43.8%)	N/A
*Comorbidities—n (%)*							
Smoking history*	18 (17.6%)	11 (21.6%)	7 (13.7%)	10 (38.5%)	1 (10.0%)	9 (56.3%)	0 (0%)
Myocardial infarction*	11 (10.8%)	7 (13.7%)	4 (7.4%)	8 (30.8%)	2 (20.0%)	6 (37.5%)	0 (0%)
Congestive heart failure*	4 (3.9%)	2 (3.9%)	2 (3.9%)	4 (15.4%)	1 (10.0%)	3 (18.8%)	0 (0%)
Peripheral vascular disease*	1 (0.98%)	1 (1.9%)	0 (0%)	4 (15.4%)	1 (10.0%)	3 (18.8%)	0 (0%)
Cerebrovascular accident*	2 (1.9%)	1 (1.9%)	1 (1.9%)	3 (11.5%)	1 (10.0%)	2 (12.5%)	0 (0%)
Dementia	6 (5.9%)	4 (7.8%)	2 (3.9%)	1 (3.8%)	0 (0%)	1 (6.3%)	0 (0%)
Pulmonary disease	21 (20.6%)	10 (19.6%)	11 (21.5%)	4 (15.4%)	2 (20.0%)	2 (12.5%)	0 (0%)
Rheumatic disease	3 (2.9%)	3 (5.9%)	0 (0%)	0 (0%)	0 (0%)	0 (0%)	0 (0%)
Peptic ulcer disease	1 (0.98%)	1 (1.9%)	0 (0%)	0 (0%)	0 (0%)	0 (0%)	0 (0%)
Diabetes mellitus	36 (35.3%)	15 (29.4%)	21 (41.2%)	6 (23.1%)	2 (20.0%)	4 (25.0%)	0 (0%)
Renal disease	11 (10.8%)	4 (7.8%)	7 (13.7%)	5 (19.2%)	2 (20.0%)	3 (18.8%)	0 (0%)
Cancer (solid)	4 (3.9%)	1 (1.9%)	3 (5.9%)	2 (7.7%)	0 (0%)	2 (12.5%)	0 (0%)
HIV/AIDS	2 (1.9%)	1 (1.9%)	1 (1.9%)	0 (0%)	0 (0%)	0 (0%)	0 (0%)

*Indicates a significant difference between COVID-19 and non-COVID-19 hospitalized groups. ^+^Indicates a significant difference between COVID-19 and healthy control groups

### DNA methylation in blood is altered in COVID-19 patients

**Fig. 2:**
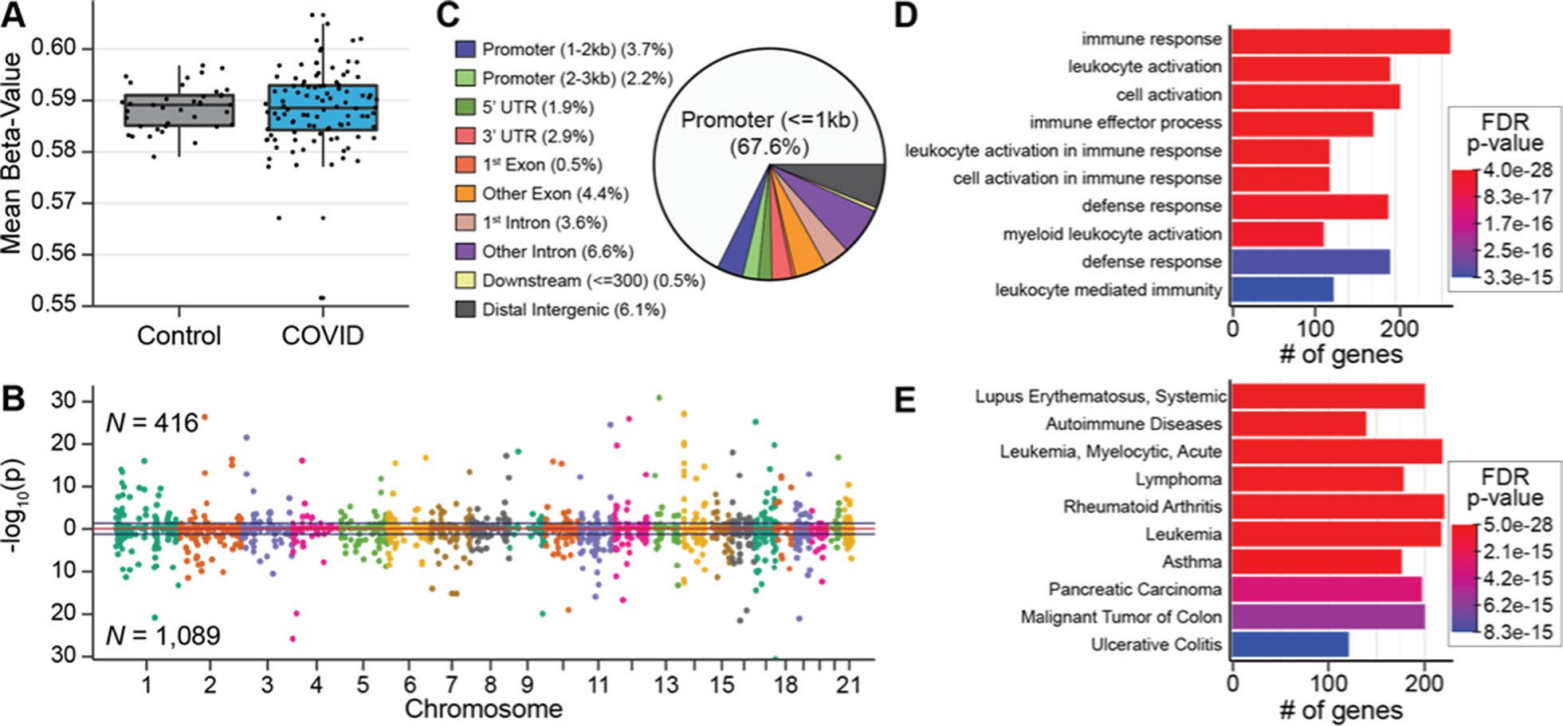
Differential SARS-CoV-2 DNA methylation between blood samples from patients on hospital admission for COVID-19 compared to blood samples from healthy controls before the COVID-19 pandemic. **A** A box and whisker plot depicts the difference in mean global methylation level (*y*-axis) between COVID-19 patients and healthy controls (*x*-axis). Each black dot represents the mean methylation level of each participant. These results indicate that global mean methylation levels do not distinguish COVID-19 patients from healthy pre-pandemic controls. **B** A Manhattan plot of DNA methylation regions shows the distribution of SARS-CoV-2-associated significantly differentially methylated regions (DMRs) across the genome by chromosome number. Hyper-methylated regions are displayed with a positive log10 (*p* value), and hypo-methylated regions are displayed with a negative log10 (*p* value). DMRs were ascertained as regions having at least 5 consecutive CpGs where > 75% of the CpGs in the region had an FDR *p* value < 0.05, and all were either hyper-methylated or hypo-methylated. This approach identified 1505 DMRs, that are displayed above and below the blue lines. Dots alternate colors to depict a change in chromosome. Sex chromosomes were excluded from analysis. These results indicate that 1505 DNA regions are differentially methylated within days of SARS-CoV-2 infection. **C** A pie chart showing the percent distribution of DMRs to standard genomic features. 5′UTR = 5′ untranslated region 3′UTR = 3′ untranslated region. In keeping with the known role of DNA methylation in regulation of gene expression, a preponderance of DMRs are in gene promoter regions. **D** Bar graphs of the top ten gene ontological (GO) biological processes related to the COVID-19 differentially methylated genes, ordered by statistical significance. The X-axis indicates the number of COVID-19 DMR-associated genes that contribute to each GO term. Bar color indicates the FDR *P*-value using a Fischer test. These results indicate that the observed DMRs occur in genes that participate in leukocyte activation and immune responses. **E** Bar Graph of the top 10 disease ontological (DO) processes related to the COVID-19-associated differentially methylated genes, ordered by statistical significance. The X-axis indicates the number of COVID-19 DMR-associated genes contributing to each GO term. Bar color indicates the FDR *P*-value using a Fischer test. These results indicate that the observed DMRs occur in genes that participate in the pathogenesis of inflammatory and leukocyte disorders

Average DNA methylation abundance across the entire genome did not significantly differ between COVID-19 patients (58.8%) and healthy pre-pandemic controls (58.7%), indicating that no global changes in methylation abundance are related to COVID-19 (Fig. 2A). To investigate locus-specific DNA methylation levels linked to COVID-19, methylome data were subjected to a linear regression model that accounted for batch effects, sex, and leukocyte proportions for downstream analyses [43]. This approach detected 1505 differentially methylated regions (DMRs) distributed across the entire genome comprising clusters of ≥ 5 CpGs (FDR *p* value < 0.05; Fig. 2B; Additional file 1: Table S2-1). A total of 416 hyper-methylated and 1089 hypo-methylated DMRs were distinguished, indicating that a majority of differentially methylated regions are hypo-methylated, as noted in a recent report of 7 COVID-19 positive patients [10]. A majority of DMRs (~ 75%) reside within or near gene promoter regions, denoting a potential role in gene regulation [16] (Fig. 2C). The 1,505 DMRs were annotated to 1,680 unique genes, indicating that several DMRs spanned two contiguous genes that harbor alterations in DNA methylation in the presence of SARS-CoV-2 infection. To test the relationships between the DMR-associated genes, we conducted a gene ontological analysis and found significant enrichments of immune-related terms, including immune responsivity, leukocyte activation, and defense responses, together with a diversity of recognized immune function genes (cytokines/chemokines and receptors (including *IL-10*, *IL-1β*, *CXCR2/5/6*), interferon-stimulated genes (*IFIT3*, *ISG20*), and signal transduction genes (*TRAF2*, *ZAP70*), (FDR *p* value < 0.05; Fig. 2D; Additional file 1: Table S2-2). A disease ontological analysis of methylation regions that differ between COVID-19 patients and healthy pre-pandemic controls indicated significant associations of DMR-associated genes with autoimmune diseases, including systemic lupus erythematosus and rheumatoid arthritis (FDR *p* value < 0.05; Fig. 2E; Additional file 1: Table S2-1). We observed no difference in the gap between chronologic age and “epigenetic clock” age between COVID-19 patients and healthy pre-pandemic controls, suggesting that there is no difference between the two groups in predisposition and resilience to an acute infection known to have enhanced severity in the elderly [44, 45] (Additional file 2: Figure 1). These findings indicate that differential patterns of COVID-19 DNA methylation in blood occur in the promoter regions of immune-related genes.

### DNA methylation in blood is specific to SARS-CoV-2 infection

**Fig. 3:**
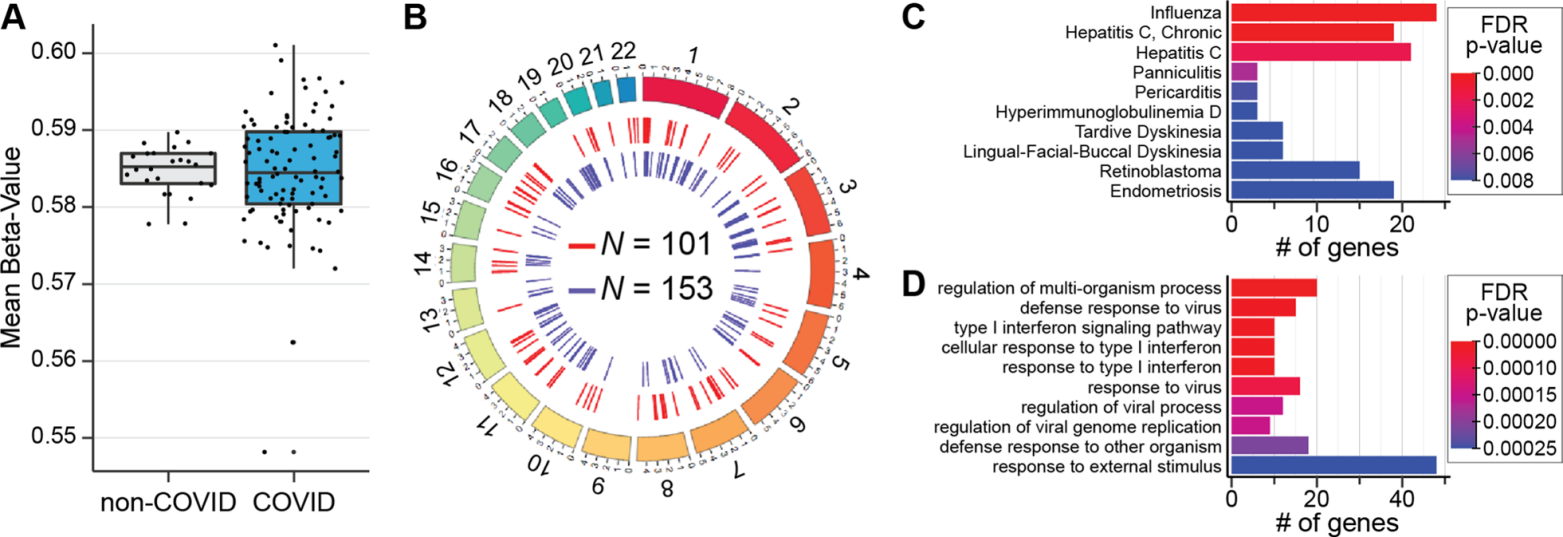
DMRs in blood samples from COVID-19 patients on hospital admission are distinct from patients with non-COVID-19 respiratory illness in genes that participate in virus-related pathways and disorders. **A** Box and whisker plot depicts the difference in mean global methylation level (*y*-axis) between COVID-19 and non-COVID-19 respiratory ill patients (1 and 0, respectively; *x*-axis). Each black dot represents the mean methylation level of each participant. These results indicate that global mean methylation levels do not distinguish COVID-19 from non-COVID-19 respiratory ill patients. **B** Circos plot depicts genomic distribution of differentially methylated regions (DMRs) across the human genome. (Outer ring) Each chromosome is shown as a different color. The relative chromosome size is represented by the arc bar length. (Inner rings) Hyper-methylated DMRs are shown in red and hypo-methylated regions are shown in blue. Sex chromosomes were omitted from the analysis. These results indicate that 254 DNA differentially methylated regions distinguish SARS-Cov-2 infection from non-COVID-19 respiratory illness. **C** Bar Graph of the top ten disease ontological (DO) biological processes related to the SARS-CoV-2-associted differentially methylated genes, ordered by statistical significance. The *X*-axis indicates the number of SARS-CoV-2 DMR-associated genes that contribute to each DO term. Bar color indicates the FDR *p* value using a Fischer test. These results indicate that the observed DMRs occur in genes that participate in inflammatory and host-defense processes. **D** Bar Graph of the top ten gene ontological (GO) processes related to the SARS-CoV-2-associated differentially methylated genes, ordered by statistical significance. The *X*-axis indicates the number of SARS-CoV-2 DMR-associated genes that contribute to each GO term. BAR color indicates the FDR *P*-value by using a Fischer test. These results indicate that the observed DMRs occur in genes that participate in the pathology of influenza, other viral infections and inflammatory disorders

To identify DNA methylation profiles that distinguish concurrently enrolled respiratory patients with and without COVID-19, we analyzed data of 128 patients, with (*N* = 102) and without (*N* = 26) COVID-19 diagnosis collected concurrently at Albany Medical Center (Fig. 1, Table 1). Four samples (two COVID-19 and two non-COVID-19 patients) were removed due to unreliable methylation values (Fig. 1) and 95,447 probes were removed leaving 770,412 for further analysis. Average DNA methylation abundance across the entire genome did not significantly differ between groups (COVID-19 patients: 58.5%; non-COVID-19 patients: 58.4%, Fig. 3A), indicating no global changes in methylation abundance related to COVID-19 status. To investigate locus-specific DNA methylation levels linked to SARS-CoV-2 infection, methylome data were subjected to a linear regression model that accounted for age, sex, and leukocyte proportions for downstream analyses [43]. This approach detected 254 DMRs distributed across the entire genome comprising clusters of ≥ 5 CpGs (FDR *p* value < 0.05; Fig. 3B; Additional file 1: Table S3-1). A total of 101 hyper-methylated and 153 hypo-methylated DMRs were identified, indicating that COVID-19 patients demonstrate changes in specific DNA positions even when compared to patients with acute respiratory decompensation due to other causes. Mapping the 254 DMRs identified 230 annotated genes, including known immune function genes (e.g., *IRF7, BCL6, MX1,* and *TNF*). A gene ontological analysis identified significant enrichment of immune-related terms, including defense response to viruses, type I interferon signaling pathway constituents, and regulation of viral genome replication (FDR *p* value < 0.05; Fig. 3C; Additional file 1: Table S3-2). Disease ontological terms disclosed significant links to other virus-causing diseases, including influenza and hepatitis C (FDR *p* value < 0.05; Fig. 2D; Additional file 1: Table S3-3). These findings indicate that COVID-19 patients demonstrate an altered blood methylome compared to that of patients with respiratory illness arising from other causes, and that differences in DNA methylation occur at genes specific to COVID-19.

### COVID-19 DNA methylation in blood and interferon-stimulated gene (ISG) expression

**Fig. 4:**
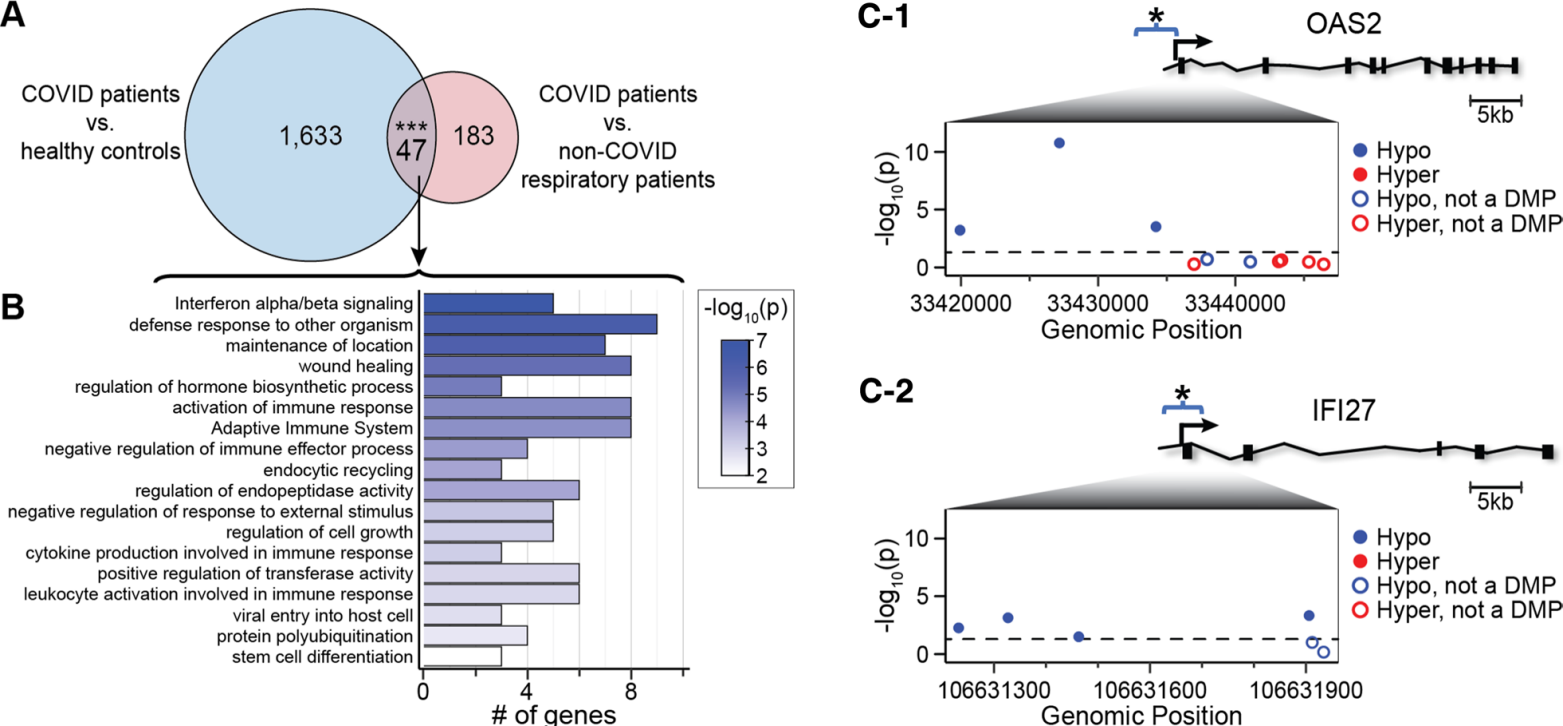
Overlap of COVID-19 DMR-associated genes in blood. **A** Venn diagram of the overlap of COVID-19 DMR-associated genes identified by comparison of DMRs between COVID-19 patients and healthy pre-pandemic controls, and DMRs between COVID-19 and non-COVID-19 respiratory illness patients on admission. Asterisks indicate overlap that is significant at *p* value < 0.001. Twenty-five of the 47 overlapping genes with DMRs encode proteins that participate in leukocyte viral defense, inflammation and immune responses. **B** Ontology analysis of the 47 overlapping genes with DMRs indicate a role in viral defense mechanisms. **C** Relative positions of COVID-19-associated DMRs in the promoter region of *OAS2* (C-1) and *IFI27* (C-2) with a schematic depicted for each gene. The relative positions of probes measuring methylation levels of CpG sites annotated to each gene with their genomic 5′-3′ positions are provided (inset panel; *x*-axis) versus the -log10 of the *p* value (*y*-axis). The *p* value < 0.05 is displayed as a black dashed line. Probes residing in a COVID-19-associated DMR are shown as hypo-methylation (blue dots) and hyper-methylation (red dots). Probes not meeting a *p* value < 0.05 at the individual CpG level are shown as hollow dots. These results indicate that the DMRs comprise a cluster of differentially methylated positions within days of SARS-CoV-2 infection

**Fig. 5:**
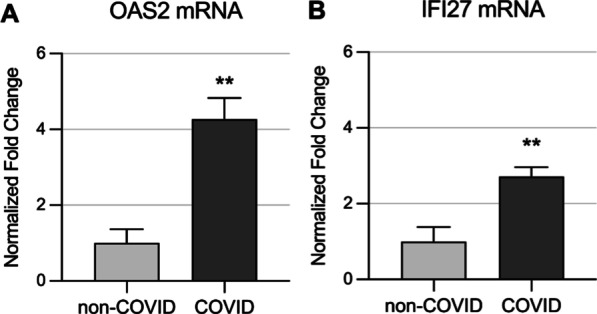
Transcriptional expression of prototypical interferon stimulated genes (ISGs) -*IFI27* and *OAS2*- correlates with methylation status of their gene promoter regions. RNA from circulating leukocytes obtained from the same COVID-19 positive and negative patients presented in Fig. 4 was used to interrogate expression level of two ISGs. **A** *OAS2* and **B** *IFI27* expression levels are significantly higher in hospitalized patients with COVID-19, which correlates with their gene promoter regions predominant hypomethylation. *GAPDH* was used as a reference gene; see methods for details. **; *p* value < 0.01

To narrow our focus on COVID-19 specific DMRs, we identified common DMRs from COVID-19 patients *vs*. healthy pre-pandemic control individuals, and DMRs from COVID-19 patients *vs*. patients with non-COVID-19 respiratory illness. Forty-seven DMRs are shared between the two datasets (Fig. 4A; Additional file 1: Table S4-1). Twenty-five of the 47 DMRs are closely linked to B lymphocyte, T lymphocyte, macrophage, and neutrophil functions, including antiviral activity, cytokine production, inflammation, and innate and adaptive immunity (Additional file 1: Table S4-2). Gene ontology and pathway enrichment analysis revealed significant enrichment in terms related to host defense responses including interferon alpha and beta signaling, defense response to organisms, and activation of the immune system (Fig. 4B). DMRs were hypo-methylated in promoter regions and contiguous sites in 2 prototypical interferon-stimulated genes, *IFI27* and *OAS2*, (Fig. 4C, D), suggesting possible regulatory effects on gene expression. Both previously published RNA sequencing analysis of the same samples [11], and RT-qPCR experiments done for this project confirm that transcriptional products of *IFI27* and *OAS2* are upregulated in COVID-19 samples in comparison with non-COVID-19 control patients (Fig. 5A, B).

To gain insight into the effects of DMRs on gene expression, we compared DMRs between COVID-19 patients and patients with non-COVID-19 respiratory illness, with differentially expressed genes (DEGs) identified in our RNAseq analysis of circulating leukocytes from the same patients [11]. We identified 36 genes that were both differentially methylated and differentially expressed in COVID-19 patients. This gene set was highly enriched in the gene ontology term: defense response to virus (27/36 genes) and a Reactome gene set: interferon signaling (19/36 genes) (Additional file 1: Tables S5-1 and S5-2). Eight in the interferon pathway were upregulated in parallel with the presence of DMRs in their genes. All identified DMRs were hypo-methylated with at least 5 consecutive CpGs near promoter regions (Additional file 1: Table S5-1).

### DNA methylation in blood and COVID-19 severity

**Fig. 6:**
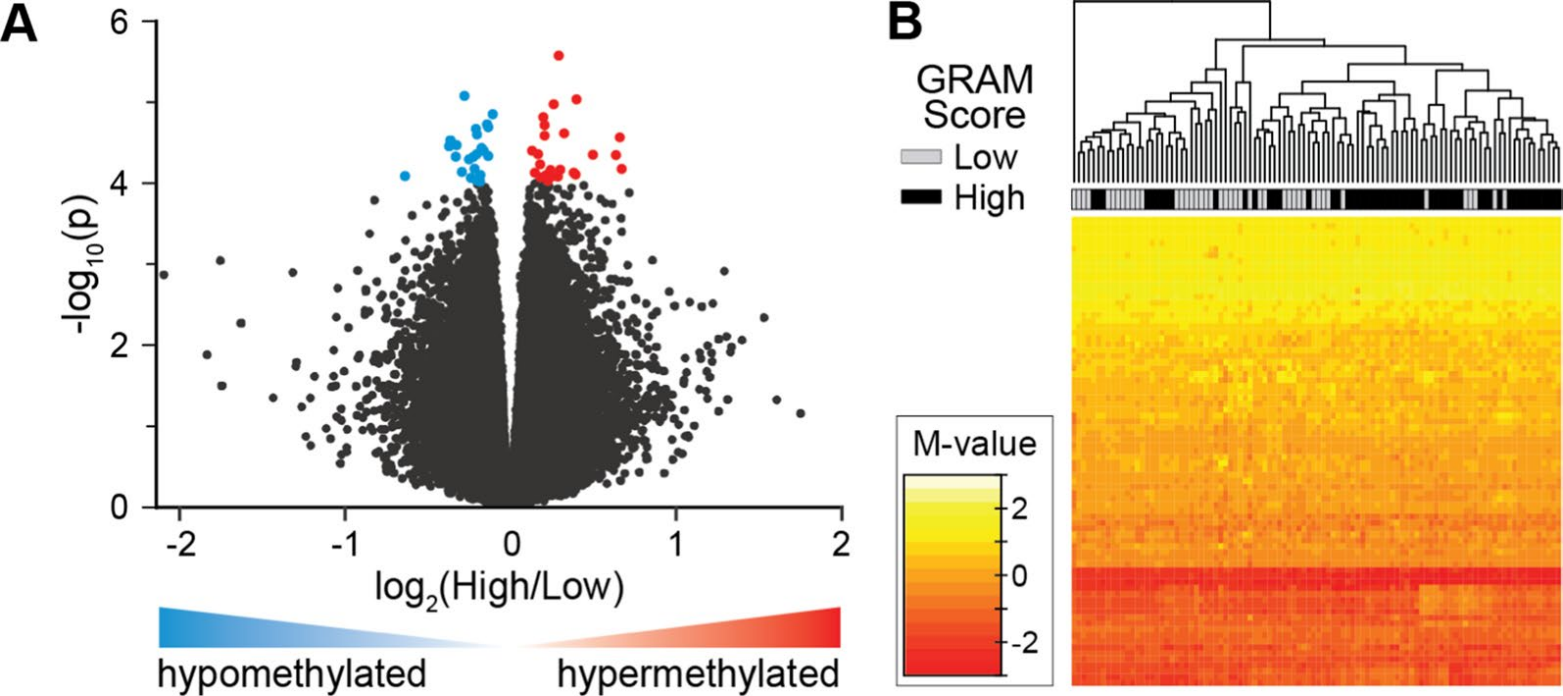
DNA methylation is associated with COVID-19 outcomes. **A** Volcano plot shows genes associated with dichotomized GRAM-risk scores, either hyper-methylated (red) or hypo-methylated (blue). **B** DNA methylation levels at 77 differentially methylated positions (DMPs) correlate with disease severity in COVID-19 patients. DMRs (*N* = 19) associated with the GRAM-score were identified in COVID-19 patients (*N* = 100). DMRs were ascertained as regions with at least 3 consecutive CpGs where > 75% of the CpGs in the region had a FDR *p* value < 0.05 and all were either hyper-methylated or hypo-methylated. DNA methylation levels of the DMPs (*N* = 145) residing in the DMRs were subjected to recursive feature elimination to identify CpGs that best distinguish GRAM-score risk. Shown is a hierarchical cluster using the DNA methylation data from the 77 DMPS (see Additional file 1: Table S8), that are shown as a heatmap of the M-values. Low GRAM-score risk (gray) and high GRAM-score risk (black) are indicated. These results indicate that DNA methylation levels at these 77 DMPs may be useful as biomarkers of the severity of COVID-19 patients*.* (see Additional file 1: Table S6-1 and S6-2)

The GRAM score is a validated outcome measure that defines the risk of deterioration in COVID-19 patients [40]. We obtained GRAM scores and mortality outcomes in our cohort, which allowed comparison of different disease burdens with DMRs in blood, and to test the potential value of DMR analysis as a predictor of patient prognosis. The GRAM-score risk percentage was dichotomized into a discrete variable (i.e., low [< 50%] and high [> 50%]) and DNA methylation data was regressed on this variable in the COVID-19 respiratory patients (*N* = 100). Because the GRAM-risk score has been validated for specific use in COVID-19 patients [40], only patients with COVID-19 were included in the analysis (Table 1). Nineteen DMRs with ≥ 3 consecutive differentially methylated CpGs were identified, (*p* value < 0.0001, Additional file 1: Table S7) between patients with low and high GRAM-risk scores. In total, the DMRs comprised 145 differentially methylated positions (DMPs), of which there were 84% located at gene promoter regions and ~ 65% were hyper-methylated (Fig. 6A). Evaluation of mortality as an outcome measure identified 18 DMRs comprising 113 DMPs, 62% of which were hyper-methylated.

To identify specific DMPs that best define GRAM-score risk, the DNA methylation levels at these 145 GRAM-risk score-associated DMPs were subjected to a recursive feature elimination analysis [46]. This algorithm revealed 77 DMPs with methylation levels that distinguish COVID-19 severity in a hierarchical cluster analysis (Fig. 6B). These data suggest that worse outcomes are associated with hyper-methylation in promoter regions and that specific positions throughout the genome may potentially correlate with COVID-19 severity.

## Discussion

In this prospective cohort study, we tested the hypothesis that COVID-19 patients demonstrate patterns of DNA methylation in blood that are different from pre-pandemic healthy individuals, and from patients with respiratory illness who did not have COVID-19. We also tested whether worse outcomes in COVID-19 patients were associated with DMRs and DMPs in blood.

### DNA methylation in blood altered in COVID-19 patients

In samples obtained within days of acute SARS-CoV-2 infection, patients exhibit 1089 (72%) hypo-methylated regions and 416 (28%) hyper-methylated regions comprising 5 or more consecutive differentially methylated CpGs in comparison with healthy control blood samples collected before the COVID-19 pandemic (Fig. 2B). A recent report comparing patients with and without sepsis of unspecified origin indicates differential methylation at genes that participate in interferon-gamma-mediated (IFNγ) signaling, MHCII antigen processing and presentation, immunoglobulin production, and cell adhesion pathways [47]. In a limited study of 6 patients with SARS-CoV-2 infection, 6 DMPs (not DMRs) were observed in genes that encode proteins that participate in granulopoiesis and B-lymphocyte-to-granulocyte trans-differentiation [10]; and a very recent report of a larger cohort identifies 44 CpGs in 39 genes that were differentially methylated, including genes related to interferon response to viral infections [48]. It has also been previously reported that viral infections induce aberrant methylation patterns in host cells [33, 49]. For instance, H5N1 influenza and Middle Eastern respiratory syndrome coronavirus (MERS-CoV) infections down-regulate interferon-stimulated and antigen-presenting genes, which are associated with hyper-methylation of gene promoter regions in human airway epithelial cells in vitro [28, 29]. The large number of DMRs identified by the very conservative criteria and inferential comparisons used here, and the diversity of their corresponding loci and pathways, are surprising in view of the short interval from infection to hospitalization in the enrolled patients, thereby potentially denoting the role of the methylome as a rapid responder to SARS-CoV-2 infection. Interestingly, a very recent report focused on pediatric critical illness demonstrates a rapid regulation of DNA methylation in circulating leukocytes, taking place within the first three days of hospitalization [50].

Genes comprising DMRs between patients with COVID-19 and healthy pre-pandemic controls include IFN-stimulated genes (ISGs), with well-recognized antiviral activity such as *IFI27* and *OAS2*. Differential methylation of type I IFN pathway genes in specific leukocyte subsets is associated with autoimmune disorders including Sjogren’s syndrome, Lupus, Grave’s disease, and rheumatoid arthritis [51–55], indicating a possible role for ISG methylation in the dysregulation of inflammatory processes, and autoimmunity as a potential contributor to COVID-19 pathogenesis [56, 57]. Much less is known about the impact of ISG methylation in blood on the control of viral infections. Recently, a correlation between ISG methylation and the outcome of HIV infection has been reported, with hyper-methylation of interferon and antiviral genes correlated with improved HIV control [58]. In SARS-CoV-2 infection, differential methylation and expression of antiviral ISGs may influence viral replication and spread in leukocyte subsets [59], or contribute to COVID-19 pathogenesis by altering immune cell activation or function. Multiple DMRs reported here appear in genes recently found with dysregulated expression levels in samples from the identical patients [11]. As previously described, methylation of CpGs located at promoter regions is canonically associated with transcriptional repression [16]. Mechanistically, methylated CpGs recruit complexes holding methyl-CpG binding domain-containing proteins and other factors that aggregate into multiprotein repressive complexes to silence transcription [60, 61]. Of note, while our previously reported RNA sequencing analysis [11] and the qPCR experiments presented here indicate that expression of ISGs, IFI27 and OAS2 is upregulated in COVID-19 patients, their differential hypo-methylation in gene promoter regions suggests that methylation may contribute to their transcriptional regulation. Future studies focused on cross-ome trajectory analyses combined with locus-specific interrogation of DNA methylation will add clarity to this possibility.

### DNA methylation in blood and COVID-19 severity

To determine if disease severity in COVID-19 patients is associated with DMRs in blood, we tested the association of DMRs with clinical outcomes including the GRAM risk score [40] and mortality. We found that worse GRAM scores were associated with 19 DMRs comprising 145 differentially methylated positions (DMPs) in 18 genes. Sixty-three percent of the GRAM-score-associated DMPs were hyper-methylated. Mortality was associated with 18 DMRs comprising 113 DMPs in 17 genes (Additional file 1: Table S9). In this setting, 61% of the DMRs were hyper-methylated. Over 84% of the DMRs associated with outcomes were located in gene promoter regions; notably, promoter hyper-methylation is canonically associated with transcriptional repression [15, 16, 18]. Previous research indicates that non-permissive (immunosuppressive) transcriptomic states are associated with worse outcomes in critical illness [30, 62–64]. Moreover, protracted COVID-19 is associated with blockade of T-cells proliferation [65] and suppression of the innate immune system in circulating blood [13]. These data suggest that as COVID-19 severity increases, promoter-predominant hypermethylation may regulate transcriptional repression at critical genes, potentially influencing the pathophysiology of host response. Future investigations with animal models will enable further elucidation of these pathways, and test whether promoter hypermethylation induced by greater severity downregulates gene expression and is physiologically consequential.

Using recursive feature elimination, we identified 77 DMPs that discriminate clinical outcomes. Given the observed epigenetic changes were captured at the time of patients’ enrollment, their combined use with other biochemical variables may support an accurate predictive instrument to guide care before clinical deterioration. This instrument may be of value for resource-allocation in a pandemic environment with an overwhelmed healthcare system [66]. Future studies based on whole genome methylation sequencing will provide the opportunity to capture other outcome-associated DMRs and DMPs and provide a more comprehensive instrument based on a shared principle [11, 67, 68].

### Strengths and limitations of this study

While the global RNA transcriptomic profiles in blood have been previously reported in sepsis [30], acute respiratory distress syndrome [31, 69] and COVID-19 [10, 11, 13], and there are recently reported small cohorts describing blood DNA methylation in COVID-19 [70], there are no prior reports that compare differentially methylated regions blood samples from COVID-19 patients to samples collected before the SARS-CoV-2 pandemic using a shared epigenotyping platform. Together, DNA methylation and RNA expression data may facilitate improved COVID-19 diagnosis, prognosis, and targeted treatments [71]. As well, we provide a first large, prospective cohort study that addresses the association of COVID-19 blood methylome and patient-centered outcomes including mortality. Future studies will investigate the association of DMRs in blood with longer-term outcomes. For example, COVID-19-induced DMRs may persist long after acute care, contributing to the post-ICU syndrome comprising physical and cognitive dysfunction [72–75]. Recent data indicate that blood DNA methylation profiles mediate worse neurocognitive development in the pediatric ICU population [25], which could be relevant in COVID-19 as well [76, 77].

Our study has some limitations. First, COVID-19 patients were enrolled in a single center. Although our population is racially diverse, it does not necessarily replicate factors related to geography or population socioeconomic status elsewhere. Second, while we enrolled the patients near the time of hospital admission, we did not control the interval that elapsed between disease onset and the blood sampling (Table 1). While most observational clinical ICU research is based on inception cohort studies in which the timeline is arbitrarily defined by the moment of patient enrollment [78], the time elapsed between admission and enrollment could have an effect on the analyzed data. Of note, previous research on the transcriptome of patients with sepsis indicated that the timing of blood sampling in relation to ICU admission was not predictive of the patients’ expression profiles [30]. Third, due to the urgency-driven pandemic environment and the initial lack of formal recommendations by medical society guidelines, we had no control of the various drugs administered to the patients including azithromycin, hydroxychloroquine, corticosteroids and others, which could have impacted the overall COVID-19 data generated. Fourth, our study relies on a cross-sectional blood sample per patient on admission and must be complemented with longitudinal sampling and trajectory analysis to ascertain the dynamics of DNA methylation in COVID-19. Lastly, our analysis is based on a high-capacity chip array which despite contributing valuable information, is limited to about 3.4% of the genome [79]. Future studies based on whole methylome sequencing analysis will assure a more highly resolved database of DMRs associated with COVID-19 severity.

In summary, we generated pre- and post-COVID-19 methylome maps, and have shown that while acute respiratory disease causes substantial changes in the DNA methylation status leading to a predominantly hypomethylated state, COVID-19 infection is associated with differentially methylated regions impacting, among others, IFN-stimulated genes (ISGs). Using RNA sequencing analysis from the same patients [11], and confirmed with qPCR experiments to interrogate two prototypal ISGs, we found that gene promoter regions of over-expressed transcripts are hypomethylated in COVID-19 versus non-COVID-19 patients, thereby suggesting an epigenetic regulatory mechanism played by CpG methylation. Moreover, we found that sicker patients exhibit a predominantly promoter hypermethylated profile, possibly suggesting a regulatory role of DMRs in the immunosuppressive gene profile already described in severe COVID-19 cases [13]. Finally, using a recursive feature elimination algorithm, we identified a limited number of DMPs that accurately discriminate clinical outcomes, suggestive the potential role of DNA methylation profiling in the early prognostication of COVID-19 patients.

### Methodological considerations

The use of the Illumina Infinium MethylationEPIC 850,000 BeadChip facilitates comparisons of data between investigations that employ a shared platform comprising sites that span the genome. This approach, which predominantly captures circulating leukocytes DNA, has been recently used in the intensive care setting [25]. However, the array is intrinsically biased by a priori selection of regions targeted for interrogation and does not incorporate over 24,000,000 additional CpGs amenable to direct sequencing of the entire methylome. While use of mixed cell populations in whole blood is of high relevance in infectious disease diagnosis and prognosis [67], and has supported identification of actionable sub-phenotypes [34, 35, 71], it does not capture processes taking place in other tissue compartments or specific cell types relevant to the pathogenesis of the COVID-19 that may arise from tissue or cell type-specific DMRs.

Whereas the nucleotide sequence of the genome is remarkably stable from conception to death [15, 16], our data contribute to the awareness that DNA methylation is rapidly dynamic, influences the expression of genes that regulate COVID-19 progression [11], and potentially modifiable by acute insults which could be reversed by targeted interventions [80].

## Methods

### Cohort characteristics

#### Human subject enrollment

##### Albany Medical Center

With approval of the Albany Medical College Committee on Research Involving Human Subjects (AMC IRB Study No. 5670–20), we conducted a single-center observational study of adult subjects admitted to either the medical floor or the medical intensive care unit (MICU) of Albany Medical Center in Albany, NY. Enrollment took place between April 6, 2020, and May 1, 2020, and follow-up continued until June 15, 2020. Patients were eligible for enrollment if they were older than 18 years and were admitted to the hospital for symptoms compatible with COVID-19. Exclusion criteria were imminent death or inability to provide consent, which was obtained from the patient or a legally authorized representative. Patients were assigned to the COVID-19 group only after receiving a positive test result via nasopharyngeal swab testing using the Abbott Realti*me* SARS-CoV-2 Assay® (Abbott, IL). SARS-CoV-2 test negative participants were assigned to the non-COVID-19 respiratory patient group as controls. The cause of respiratory distress in the non-COVID-19 patients is presented in Additional file 1: Table S1. Pre-hospital co-morbidities determined using clinical history and hospital documentation were aggregated using the Charlson comorbidity index [42]. APACHE II, SOFA, and SAPS II scores were used to assess severity of critical illness on ICU admission [41]. Sex, age, and other relevant subject data are provided in Table 1 and the Additional file 1: Table S1.

##### Wisconsin Alzheimer’s disease research center

With approval of the University of Wisconsin Institutional Review Board (UW IRB Study No. 2015-0300), blood samples were collected before 2017 from 39 healthy normal control participants. Participants were recruited from the community by advertisements and outreach events, and served as healthy normal controls in a Wisconsin Alzheimer’s Disease Research Center (WADRC) investigation [21]. The healthy normal control participants complete a yearly study visit consisting of a blood draw, medical history questionnaires, psychometric testing, a physical exam, and must have no known diseases that interfere with study participation over time. Demographic details of the healthy normal control participants are provided in Table 1.

#### Selection of outcome measures

We analyzed the data with an outcome measure that: (1) is able to combine the severity of disease with mortality in a single metric; (2) is applicable to both ICU and medical floor populations; (3) uses a timeframe that accounts for longer hospitalizations in COVID-19 patients with respiratory failure compared with non-COVID-19 individuals [3, 81]; (4) accounts for COVID-19 linear deterioration that transitions from mild respiratory compromise to respiratory failure, followed by respiratory distress requiring mechanical ventilatory support and eventually death. Thus, we selected the composite outcome variable defined by the COVID-19 risk GRAM score [40]. Characteristics contributing to the determination of the COVID-19 risk GRAM score are shown in Additional file 1: Table S6-1 and 6-2. To simplify the analysis, patients were separated into two groups based on a calculated risk percentage below or above 50%. The secondary outcome measure was in-hospital mortality.

#### Sample collection and storage

At enrollment, blood samples were collected using BD EDTA Vacutainers^®^. Whole blood was then aliquoted and frozen at − 80 C degrees for later processing and analysis.

#### DNA isolation and methylation microarray

DNA was isolated from 500µL of frozen whole blood using the GeneJET whole blood kit (Thermo Fisher Scientific, K0782) following the manufacturer’s protocols. DNA concentration was determined using a Qubit fluorometer (Thermo Fisher Scientific) and normalized to 20 ng/µL for microarray analysis. Samples were shipped overnighted to Genuity Science Inc. (Boston, MA) for bisulfite conversion and methylation microarray analysis using the Illumina Infinium MethylationEPIC Beadchip array [82]. The shared collection and processing of the blood DNA methylation levels from the Wisconsin healthy individuals’ cohort (WADRC) was previously published [21].

#### Leukocyte messenger RNA (mRNA) expression determination

Whole blood samples were simultaneously collected from all participants, and leukocytes were isolated using LeukoLOCK filters (Cat. No. AM1923; Thermo Fisher). RNA was then extracted from the filters following the manufacturer’s instructions and as previously reported [83]. Five hundred nanograms of total RNA was used to prepare cDNA using Qiagen RT Master Mix at 42 °C (Cat. No. 210215; Qiagen) following the manufacturer’s instructions. After RT reaction, 2 µL of cDNA was used per qPCR reaction. qPCR was performed on a CFX Connect (Bio-Rad) instrument using SYBR green-based universal iTaq supermix (Cat. No. 1725125; Bio-Rad) and 2 pmol primers (IDT). Fold induction was calculated using the ΔΔCt method using GAPDH as the reference gene. Each sample was assayed in triplicate, and a negative control was included in each experiment. Primer sequences can be found in Additional file 1: Table S10.

#### Illumina human MethylationEPIC data preprocessing

To identify methylation changes associated with COVID-19, we compared COVID-19 patients (*N* = 102) to methylation data from pre-pandemic participants [21] that were enrolled 3 or years before the SARS-CoV-2 outbreak (*N* = 39). Raw.idat files from all (*N* = 141) were imported to the R environment. R package minfi was used to parse and preprocess methylation microarray data [84]. The quality of raw data was assessed, and no samples were filtered due to high mean detection *p* value (*i.e.,* mean > 0.05). Bisulfite conversion of samples was assessed for each sample by density and bean plots, and determinations, to assure that the distribution of beta-values was bimodal with the largest densities being centered on 0 or 1, and that the majority of data was either methylated or unmethylated. All samples were deemed to be successfully converted. Leukocyte proportions were estimated from methylation signatures, and cell counts were extracted for incorporation into models of differential methylation. Samples were normalized using functional normalization by background and dye correction following the normal-exponential out-of-band method [85]. Following normalization, sex prediction was generated using normalized values.

Two COVID-19 samples were removed due to improper sex prediction from the COVID-19 and non-COVID-19 cohorts each, suggesting unreliable methylation values from these samples. Probes were removed from remaining samples (*N* = 139) if any of the following criteria applied: probes measured methylation on sex chromosomes; probes contained or reported methylation at SNPs; probes measured methylation at CH sites; detection *p* value of a probe > 0.01 for at most one sample; and probes were known to be cross-reactive. This filtering approach removed 99,905 probes through quality processing, leaving 765,954 for further analysis. Beta-values and logit M-values from the remaining probe set were generated for differential analysis. A one-way ANOVA was used to determine significant differences between mean beta-values of patients between groups.

To identify methylation changes associated specifically with COVID-19 versus non-COVID-19 respiratory patients, or other variables of interest (i.e., GRAM score, and mortality), raw.idat files from the AMC cohort (*N* = 124) samples were imported to the R environment. R package minfi was used to parse and preprocess methylation microarray data [84]. The quality of raw data and bisulfite conversion were assessed, leukocyte proportions were estimated, and samples were normalized, as above. After normalization, sex prediction was generated using normalized values. Four samples were removed due to improper sex prediction, suggesting unreliable methylation values for these samples. Probes were removed from remaining samples (*N* = 124) using the criteria as above. Filtering removed 95,447 probes through quality processing, leaving 770,412 for further analysis. Beta-values and logit M-values were generated for differential analysis. A one-way ANOVA was used to determine significant differences between mean beta-values of patients between groups.

#### Model selection for differential analysis

Several potential models using available covariates were assessed to generate the best fit for the data. To compare COVID-19 (*N* = 100) samples with pre-pandemic samples (*N* = 39), models accounting for COVID-19 status (positive vs. negative), age, sex, and estimated leukocyte proportions (i.e., granulocytes, monocytes, natural killer cells, B lymphocytes, CD8 T lymphocytes, CD4 T lymphocytes) were generated. Model selection was based on BIC score criterion. Of the tested models, a model accounting for COVID-19 status, sex, and leukocyte proportions was preferential and used for downstream analyses. Batch effects between microarrays were adjusted using ComBat from the R package sva [86]. Batch-adjusted beta- and M-values were assessed by the R package sva to identify unknown confounders such as with other infections or complications. The surrogate variables found were adjusted for during model fitting.

To compare COVID-19 respiratory patients (*N* = 100) with non-COVID-19 respiratory patients (*N* = 24), the model selection was performed as above.

When assessing methylation levels associated with mortality of COVID-19 patients (*N* = 100) and GRAM score (*N* = 100), model selection was performed as above. Based on BIC criterion, models adjusted for surrogate variables using sva were selected for downstream analysis. Two outlier samples were removed from the GRAM score analysis because their scores were greater than 3 standard deviations from the mean.

#### Detection of differentially methylated regions

R package DMRcate was used for the detection of differentially methylated regions (DMRs) [43]. M-value matrices were annotated to their chromosomal position, and test statistics were generated for variables of interest using models as described above. For comparisons of COVID-19 patients versus pre-pandemic healthy participants, and COVID-19 patients versus non-COVID respiratory patients, DMRs were identified using an FDR *p* value cutoff of 0.05 and a minimum of 5 CpG sites in the region. For the comparison of methylation levels to GRAM score, and mortality, criteria for DMR identification included a *p* value cutoff of 0.0001 and a minimum of 3 CpG sites in the region. Genes annotated to DMRs were extracted for downstream ontological analyses.

#### Ontological analyses

Genes comprising DMRs were assessed for ontological analyses of biological processes and diseases using the R package clusterProfiler [87]. A listing of background genes was generated from all tested regions from DMRcate (*N* = 20,899 genes). Gene symbols were converted to ENTREZIDs. Significant terms were determined using an FDR *p* value cutoff of 0.05, comparing differentially methylated genes to the background gene list.

#### Plot generation

Manhattan plot generation used R packages qqman and ggplot2. For the pie plot, R package ChIPseeker was used to annotate regions [87]. Bar plots of ontological terms were generated using the R package clusterProfiler. Hypergeometric tests in the R environment were used to identify enrichments of gene lists. Customized Circos plots were generated using the R package BioCircos [88]. For heatmap generation of dichotomous GRAM score data, the R package caret was used for backward feature selection, starting with a matrix of M-values from all CpGs in identified DMRs from the comparison. For model selection, cross-validation methodology and 5 iterations using subsets of 1–100 CpGs were used. Heatmaps were generated using the R packages gplots and heatmap.plus.

## Supplementary Information

**Additional file 1.** Descriptions of COVID-19 related DMRs.

**Additional file 2.** Comparison of chronologic age and “epigenetic clock” age between COVID-19 patients and healthy pre-pandemic controls.

## Data Availability

The datasets generated during and/or analyzed during the current study will be publicly available as soon as a GEO accession number GSE174818 is generated.
