# Supplementary figures and images for: Blood DNA methylation and COVID-19 outcomes
Source: Clin Epigenetics. 2021 May 25;13:118. doi: 10.1186/s13148-021-01102-9 (PMC8148415; doi:10.1186/s13148-021-01102-9)

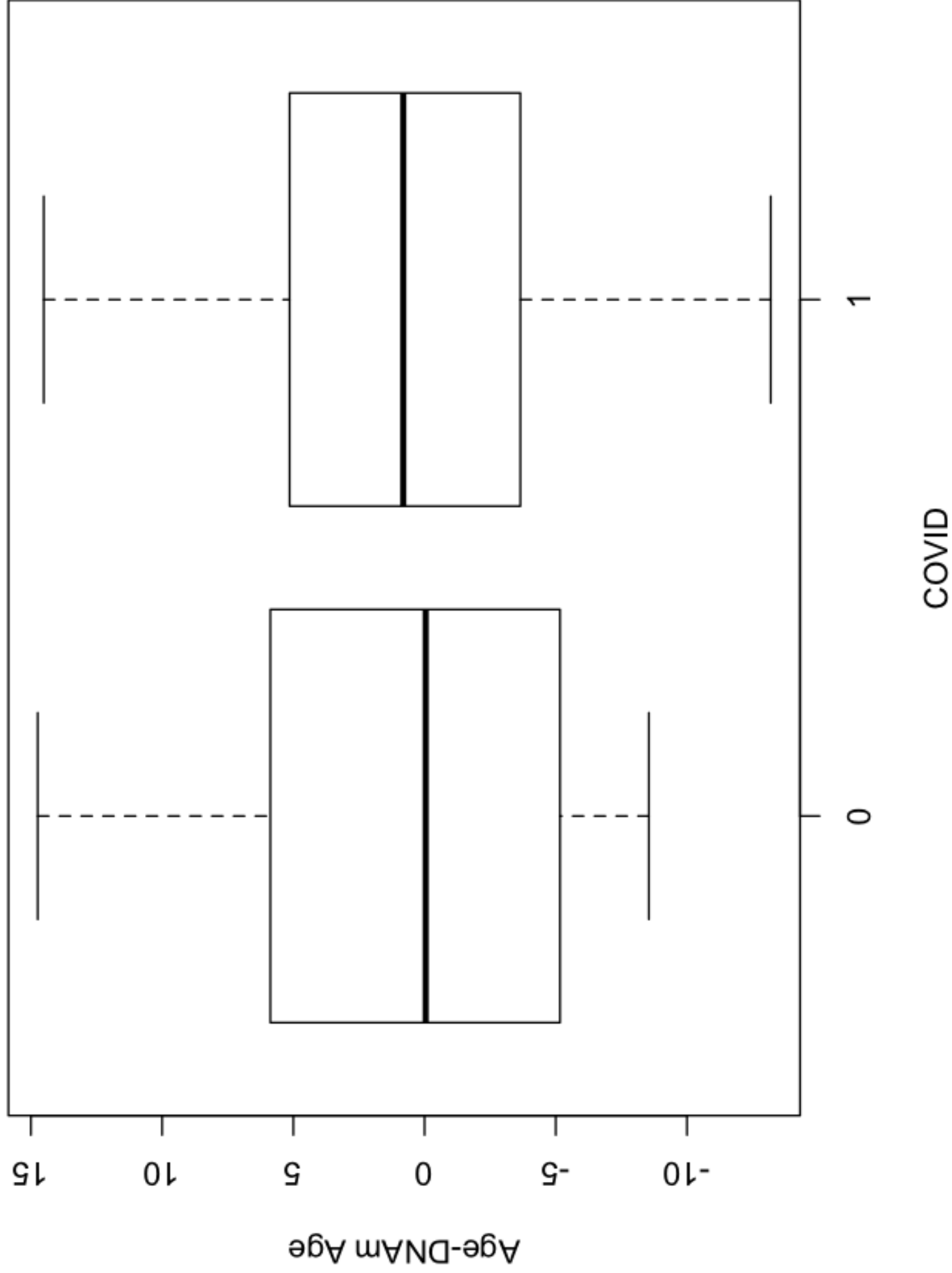

Supplement: Supplementary file 2 — Additional file 2. Comparison of chronologic age and “epigenetic clock” age between COVID-19 patients and healthy pre-pandemic controls. [file 13148_2021_1102_MOESM2_ESM.pdf]
